# Public policy (not the coronavirus) should shape what endemic means

**DOI:** 10.7189/jogh.12.03050

**Published:** 2022-08-01

**Authors:** Ronald U Mendoza, Kenneth Y Hartigan-Go, Alex B Brillantes, Karl E V Ruiz, Ivyrose S Baysic, Sheena A Valenzuela

**Affiliations:** 1 School of Government, Ateneo de Manila University, Quezon City, Philippines; 2 Eastern Regional Organization for Public Administration, Quezon City, Philippines; 3 Ateneo Policy Center, School of Government, Ateneo de Manila University, Quezon City, Philippines

In January 2021, almost one year into the pandemic, a survey of more than 100 immunologists, infectious disease researchers, and virologists working on COVID-19 revealed that most – 90 percent of respondents – forecasted that the disease is likely to become endemic as it will not be eradicated [1]. They expected it to exist in certain areas of the world for years to come, despite it being virtually eliminated in other regions, as the populations there acquire some degree of herd immunity through the combined effects of vaccination and infection. Katzourakis [2] noted that “to an epidemiologist, an endemic infection is one in which overall rates are static – not rising, not falling. More precisely, it means that the proportion of people who can get sick balances out the ‘basic reproduction number’ of the virus, the number of individuals that an infected individual would infect, assuming a population in which everyone could get sick” [2].

A disease being endemic does not mean, however, that it is not deadly [2]. In 2020, there were an estimated reported 241 million malaria cases and 627 000 malaria deaths globally [3]. Ten million were afflicted with tuberculosis that same year and 1.5 million of those sick died [4]. Katzourakis further stated that “endemic certainly does not mean that evolution has somehow tamed a pathogen so that life simply returns to ‘normal’” [2]. Hence the risk of a resurgence will likely continue as the disease may pose a seasonal risk of reintroduction into areas where vaccination and public health measures are relatively less robust.

A growing number of policymakers, such as those from Spain and the United Kingdom are calling on their people to start “living with COVID-19” and treat the disease as an endemic disease [5]. Yet there is no specific definition of what endemic means from a policy perspective. Countries are likely to define endemic based on concrete public policy actions. Based on the available science and evidence, it is useful to revisit and clarify this term, as well as begin to define its possible main elements from a policy lens and in the context of COVID-19.

## WHAT ENDEMICITY MEANS FOR PUBLIC POLICY

The main message here is that a disease becoming endemic is not simply an outcome of the inevitable evolution of the disease. Much of this is still highly uncertain when considering where the world is today with the COVID-19 pandemic. Endemicity can also be shaped by policy decisions, often with deep efficiency and equity implications. How the world (via international cooperation) and how individual nations (via social safety nets and universal health care policies) live with COVID-19 are important policy issues. Simply declaring COVID-19 “endemic” does not wipe away this governance accountability. Governance implications – in terms of participation, transparency, and accountability, coupled with the imperative to address inequities (much more than inequalities) – must simply be addressed.

We want to emphasize that endemicity is not only a biological and health event but has several inter-dependencies cross-cutting with the management of the economy, including governance and policies. Within the context of the discourse on inequity, what does endemicity mean for poor and low-income families, where policies on social inclusion and social welfare need to be re-calibrated? Imagine, for example, how the policies influencing the management of endemicity will include how schools are opened, how people travel across cities, how workers (both formal and informal) access insurance and health care, and how much (and in what form of) support firms receive to be able to manage risks by changing physical infrastructure during the “new normal”. These adjustments also involve entire families learning to manage risk – for instance, if one family member catches COVID-19, what does this mean to work and productivity and the health care costs associated with isolation and quarantine. The financial burden alone is huge for those in the lower socio-economic bracket of society who are often the least capable to adapt to shocks and also the typically marginalized in terms of health care and social safety nets.

Governance has often been associated with political and administrative decisions. However, it must be pointed out that there are other equally critical elements at play. The health science & technical governance accountability is one – at times the recommendations emanating from risk-averse health experts tend to gravitate toward lockdowns, the creation of barriers, restriction of movements out of concern, and fear of overwhelming lean and under-resourced health care systems. This is a product of perennial low prioritization of health systems strengthening before the pandemic. Moving forward, what does this mean to build forward better?

## ELEMENTS OF ENDEMICITY

Endemicity can be shaped and is not always an inevitable consequence of nature [6]. The decision, at its core, has to do with society’s preference on, among others, overall national quality of life, the degree of risk it can effectively manage and is willing to accept, and concern over its most vulnerable. In many cases due to the internationally integrated nature of the world economy, a degree of international social cohesion and a social preference for international cooperation also come into play. A clear example here is the international effort to eliminate smallpox, which until 1979, was still a global threat [7]. This disease which used to persist in several pockets in the world was finally wiped out from the “wild” through concerted international cooperation. In the case of COVID-19, the health crisis has precipitated a broader economic slowdown in many parts of the world, producing not just health costs but also social, economic, and financial disruption with possible long-term “scarring” for many countries.

**Figure:**
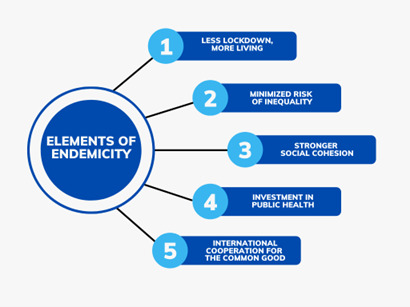
Photo: Elements of endemicity in the context of public policy (source: the authors).

It is not yet clear what the end state for COVID-19 will be, and so public policy, in confronting this wicked problem, must take a confidence-building stance amidst uncertainty. Even the scientists debate the trend in the evolution of COVID-19, with some concluding it will be difficult to model in light of the world’s uneven vaccination and health responses to the pandemic, and still, others expect risks of more virulent strains still possible [8]. While mankind cannot control nor predict how the virus will evolve, it can craft through policies a social and economic environment that brings the world closer to societies’ preferences of the “new normal”. Rather than painting this condition as an end-state, it may be more useful to illustrate it as a direction of social and economic conditions that could help bring about an acceptable degree of normalcy, as well as enhanced trust in government, public policy, and fellow citizens’ commitment to the common good.

### Less lockdown, more living

To bring about successful economic recovery, countries must be moving towards fewer mobility restrictions and certainly avoid economically costly lockdowns as much as possible. This could be possible with increased vaccination and stronger health and social protection systems that allow countries to continue to operate despite the lingering risk of COVID-19. In certain cases when a surge threatens and gets past containment systems, careful recalibration may be necessary including bringing back some mobility restrictions. A nation’s preparedness to experience this “non-linear” recovery process will be critical. A strong psychology of recovery – particularly as it relates to confidence-building in the economy – will need to be encouraged and sustained as citizens slowly re-balance their concern over the virus (and future variants) with their confidence to re-engage in social and economic life. This could not be undertaken without a coherent risk communications plan and rollout by the public sector. Government must speak clearly and credibly. Fear must be managed and trust-building made the primary goal of communication. Under these conditions, citizens are more likely to make informed choices to voluntarily follow safety protocols.

### Minimized risk of inequality

Even with hospitalization and mortality potentially declining, COVID-19 cases from future variants could still impose deep social, health, and economic costs for those most affected. The inability to contain a surge, or a policy decision to not even try, most likely redistributes costs onto the poorest households in society – those who are often most incapable of adapting to the health shock. As part of the “new normal”, countries can start moving towards expanding and deepening their social protection and health systems, making them more inclusive, nuanced, and offering support and coverage to start building up the confidence necessary to re-engage the economy despite lingering uncertainty and risks.

### Stronger social cohesion

Even if COVID-19 somehow becomes less relevant, naturally, the lesson to build these risk and health management systems as part of a build-back-better strategy for future pandemics cannot be overstated. These systems must include efforts to strengthen containment systems underpinned by strong social cohesion. When the world did not have COVID-19 vaccines yet, different countries’ efforts at containment and their ability to flatten the curve bought precious time and saved lives. But only the most effective pandemic responses managed to do this while protecting their economies from severe harm. An empirical study of 177 countries and territories found that if all countries attained at least the same level of trust in government and interpersonal trust as Denmark (notable for its relatively successful pandemic response, and in the 75th percentile of the trust spectrum across countries), then global infections could have been reduced by almost 13% due to enhanced trust in government and up to 40% due to enhanced interpersonal trust [9].

### Investment in public health

There are inequalities in social and economic conditions pre-pandemic that contributed to the high and unequal death toll from COVID-19 and must be reduced over time with the principles of equity and multisectoral engagement [10]. Because of the inter-dependent relationship between health and economy, there is cause for balance when crafting mandates. Because the economy and health are strongly linked – managing the pandemic well assists the economy to grow in the longer term, which is supportive of health; but the government must invest in public health seriously to improve response to diseases [10]. We argue that endemicity management is no different in principle. The existing instruments in countries dealing with the pandemic should not be dismantled. The human resources, technology, and equipment that were utilized for the pandemic need to be put to good use to advance health care and re-activated in case of resurgence or kept ready in response to new forms of infectious attacks.

### International cooperation for the common good and self-interest

Given the obvious scope of this global pandemic, all countries must be moving towards stronger international cooperation. It has become almost kitsch for many policymakers to quote the WHO Director-General Tedros Adhanom Ghebreyesus that “none of us are safe until we are all safe” and yet vaccine access inequality across countries and within countries continues to persist. Offering this vaccine lifeline to marginalized countries lowers the risk of new variants emerging from these global blind spots. These are crucial investments to bring about stability and normalcy, while also preventing greater inequality in the aftermath of COVID-19 [11]. The pandemic continues to test the very connectedness of international economic integration as the health crisis revealed the urgency and importance of developing some level of self-sufficiency – the production of vaccines, protective equipment, and clothing being some examples. Here it is also necessary for countries to strike a balance between further enhanced international cooperation for the global public good of inclusive and robust economic and health recovery, as well as pragmatic prioritization of country-level safeguards so that each country’s health and economic security is less exposed to supply volatility in the longer run.

### Finally, hackneyed as it may sound, governance, leadership, and trust do matter in shaping public policy

It is critical to have strong, decisive leadership. Presidents and Prime Ministers, in particular, provide leadership [12,13]. They can rally a large group of people around a set of ideas to strive for long-term objectives [14,15]. Leaders can impact a country's development [16]. Leadership is about clear communication and trust as it provides the people perspectives from a certain viewpoint, deciding what has to be accomplished, how it will be executed, and facilitating individual and collective efforts to reach shared goals [17,18].

Learning from the lessons learned over the decades, implementation has always been key to successful public policy [19,20]. Good implementation is founded upon capacities, that include strong institutions. Undergirding these institutions are distinctly defined areas of responsibilities and accountabilities, within the context inter- and intra-governmental coordination, vertically and horizontally.

Endemicity should be carefully used. Public policies must become informed by scientific evidence. The concept must not be used as a reason to be complacent, do nothing, or leave everything to chance. It may be premature for us to let our guards down. We have seen that the renowned power of modern medicine (vaccines, antiviral drugs, diagnostic testing) and a better awareness of how to halt an airborne disease through masks, distance, and air circulation and filtration are the best defences available. We need to invest in medicines that defend against a wider range of variants [2]. We must resist the mind-set of my nation first [12]. As we build up health care capacity, this pandemic has underscored that the only way out is a strong national medical system and countries receptive to multinational cooperation.

Informed, clear, and evidence-based public policies – not reactive and short-term policies, programs, and activities, that at times may be knee-jerk – must therefore define the journey towards endemicity. These would mean less lockdown and more living, addressing real inequity concerns that would lead to stronger social cohesion and more investments in public health, enabled by sound governance and accountable leadership, and supported by international cooperation for the common good.

Only then will we have an improved global public health goods system that can prepare us for the next pandemic.
